# Effect of Heat Treatment on Gradient Microstructure of AlSi10Mg Lattice Structure Manufactured by Laser Powder Bed Fusion

**DOI:** 10.3390/ma13112487

**Published:** 2020-05-29

**Authors:** Mulin Liu, Naoki Takata, Asuka Suzuki, Makoto Kobashi

**Affiliations:** Department of Materials Process Engineering, Graduate School of Engineering, Nagoya University, Furo-cho, Chikusa-ku, Nagoya 464-8603, Japan; takata.naoki@material.nagoya-u.ac.jp (N.T.); suzuki.asuka@material.nagoya-u.ac.jp (A.S.); kobashi.makoto@material.nagoya-u.ac.jp (M.K.)

**Keywords:** additive manufacturing, heat treatment process, aluminum alloy, portions of lattice structure, microstructural coarsening

## Abstract

The present study addressed the effect of heat treatment process on microstructure of an AlSi10Mg lattice structure with a body-centered cubic unit cell manufactured via laser powder bed fusion (LPBF). The as-manufactured lattice specimen exhibited a unique cellular structure composing of primary α-Al phases bounded by α-Al/Si eutectic microstructure. A gradient microstructure (continuous microstructural changes) was found in the node and strut portions composed of the lattice specimen. The microstructure appears more equiaxed and coarser with approaching the bottom surface of both portions. The continuous microstructural changes contributed to a variation in hardness measured at different locations in the as-manufactured lattice specimen. Si particles finely precipitate in the primary α-Al phases, and eutectic Si particle coarsening occurs at an elevated temperature of 300 °C. The microstructural coarsening is more pronounced at a higher temperature. A number of significantly coarsened Si particles and a stable Fe-containing intermetallic phase (β-AlFeSi) were observed at all locations in 530 °C solution-treated specimen. The homogenous microstructure results in a constant hardness value independent of the location in the lattice specimen. These results provide new insights to control the compressive properties of the AlSi10Mg lattice structure manufactured via LPBF by subsequent heat treatment processes.

## 1. Introduction

Porous metals exhibit various unique physical properties, including low volumetric density, low thermal conductivity, good permeability, high specific stiffness, and high impact energy absorption [1]. In particular, the absorption energy of lightweight porous aluminum (Al) under compression has been studied by Koza et al. [2] for potential applications in the crumple zones of automobiles. The stable plateau stress and large plateau-end strain are required to achieve high impact energy absorption. To improve the energy absorption capability, it is essential to control the structural factors of the porosity, pore shape, pore size, and pore distribution [3].

Additive manufacturing (AM) techniques enable to manufacture complicated shapes from computer-aided design (CAD) models [4,5]. Furthermore, laser powder bed fusion (LPBF) is a class of AM techniques using a laser beam to melt and fuse layers of metal powder particles forming various metal products with complex shapes, which cannot be manufactured by conventional techniques [6]. With this concept, LPBF could be a promising route to fabricate open-cell porous metals with controlled pore structures. In particular, the lattice structure (which is made up of periodic unit cells consisting of interconnected struts and nodes [7]) is a new class of porous metals. The lattice structures of Al alloys with precisely controlled porous structures could be expected as high-performance impact energy absorbers [8]. However, the lattice structures manufactured by LPBF using AlSi10Mg alloy powder (one of the most widely used Al alloys for AM [9]) exhibit unstable compression behavior with several peaks and troughs of compressive stress [10]. This compression behavior is not applicable to the structural parts of crumple zones.

Extensive studies have been carried out to evaluate the compressive properties of the LPBF-manufactured lattice structure of Al alloys. Qiu et al. assessed the effect of processing conditions on the manufacturability and compressive properties of the lattice structure [11]. The results suggested that process optimization could improve the compressive properties of the lattice structure. In addition, Delahaye et al. [12] and Maamoun et al. [13] recently reported that various process parameters could form local variation in microstructure during LPBF process, leading to microstructure defects which could influence the mechanical properties of LPBF-manufactured Al alloy as well. Amani et al. investigated the compressive deformation behavior of the lattice structure by using X-ray tomography and finite element method (FEM) [14]. Leary et al. proved that lattice geometry is the dominant factor on the compressive behavior of the lattice structure [15]. Yan et al. evaluated the relationship between compressive strength and the porosity and unit cell size of the lattice structure [16]. The compressive strength increased with decreasing the porosity or the unit cell size. Yu et al. designed various lattice structures with different unit cells and investigated the structure-property relationships in the lattice structures [17]. The designed lattice structures exhibited relatively high strength and different failure modes during the compressive test. Al-Saedi et al. manufactured a series of lattice structures with a gradually changing geometry (gradient in structural design), leading to a relatively stable deformation with a high strength [18]. Han et al. evaluated the surface roughness and dimensional accuracy of the lattice structure, which could be a factor of the compressive behavior of the lattice structure [19]. Chang et al. developed a novel electrochemical polishing process to smoothen the surfaces of the lattice structure [20]. The polished lattice structure showed high compressive stress and high energy absorption. Delroisse et al. demonstrated that the strut orientation has an effect on the microstructure, resulting in an inhomogeneous microstructure inside the inclined struts [21]. Recently, Liu et al. investigated an inhomogeneous microstructure (gradient microstructure) in the AlSi10Mg lattice structure manufactured via LPBF [22]. Furthermore, Suzuki et al. demonstrated that the heat treatments could influence the compressive behavior of the AlSi10Mg lattice structure, which significantly depends on the heat treatment temperature (300 °C and 530 °C) [23]. To optimize the compressive properties, it is required to understand a change in the gradient microstructure of the AlSi10Mg lattice structures by the heat treatments at various temperatures. The observed gradient microstructure might cause a local deformation inside the lattice structure during compression, leading to the local shear fracture. The microstructural inhomogeneity could be controlled by subsequent heat treatment, resulting in controllable compressive properties. It is therefore necessary to understand the variations in the microstructures inside the AlSi10Mg lattice structure at elevated temperatures.

Several studies have reported the effect of heat treatment on microstructure of LPBF-manufactured AlSi10Mg alloy. Li et al. investigated the effect of the solution and artificial aging heat treatments on the microstructure of the AlSi10Mg alloy [24]. Takata et al. reported the change in microstructure of the AlSi10Mg alloy with heat treatments at elevated temperatures [25]. Zhou et al. focused on the precipitates and hardness of LPBF-manufactured AlSi10Mg alloy before and after T6 heat treatment (solution treatment and subsequent aging) [26]. Maamoun et al. manufactured the AlSi10Mg alloys by LPBF from recycled powder and investigated the effect of thermal postprocessing on microstructure evaluation [27]. However, there is little information about the change in gradient microstructure of the AlSi10Mg lattice structures with heat treatments.

In this study, we have systematically characterized microstructural and crystallographic features of the LPBF-manufactured AlSi10Mg lattice structure and their changes by heat treatment process at different temperatures. These results were used to discuss the effect of homogeneous microstructure on the compressive behavior of AlSi10Mg lattice structures.

## 2. Experimental Procedure

**Figure 1:**
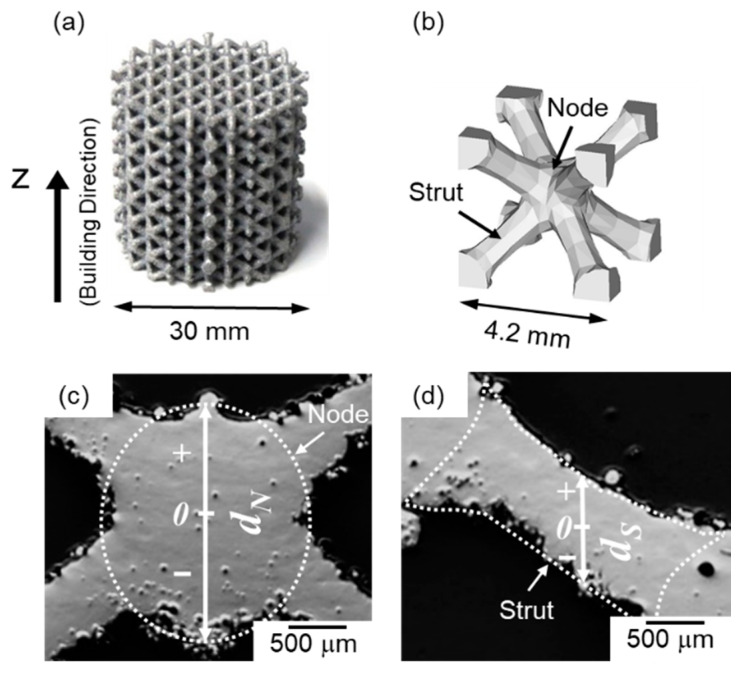
(**a**) Body-centered cubic (BCC)-type lattice structure manufactured in this study; (**b**) computer-aided design (CAD) model of BCC-type unit cell. Optical micrographs showing cross sections of (**c**) node portion and (**d**) strut portion of the BCC-type lattice specimen. The geometric centers of the node and strut portions were denoted as 0 (origins). The positive and negative Z-directions were denoted as + and −, respectively. The vertical distances from the geometric centers of the node and strut portions were designated as *d_N_* and *d_S_*, respectively.

The lattice structure specimens were manufactured with the gas-atomized AlSi10Mg alloy powder in the present study. The measured chemical composition of the alloy powder is Al–10.23Si–0.37Mg–0.12Fe (wt %) [22]. The LPBF process was carried out by using an EOS M 280 machine (EOS GmbH, Krailling, Germany) in high-purity Ar atmosphere. The A5083 aluminum base plate preheated to approximately 200 °C to reduce the residual stress of the LPBF-manufactured specimens. The detailed laser processing parameters applied to the present LPBF process are listed in Table 1. The volumetric energy density (*E*) is defined as follows,

(1) $$E=\frac{P}{v\cdot h\cdot t}$$

where *P*, *v*, *h*, and *t* represent laser power, scanning speed, hatch distance, and layer thickness, respectively. The scanning strategy was performed using a meander path, as shown in previous study [28]. The studied body-centered cubic (BCC) type lattice structure and the CAD model of the corresponding unit cell are shown in Figure 1a,b. In this study, the directions parallel to the building direction was defined as Z direction. To systematically investigate microstructures of various locations in the node and strut portions, the vertical distances from the geometric centers of the node and strut portions were designated as *d_N_* and *d_S_*, respectively. The geometric centers of the node and strut portions were denoted as 0 (origins). The positive and negative Z-directions were denoted as + and −, respectively (Figure 1c,d). The LPBF-manufactured specimens of the AlSi10Mg lattice structure were annealed at 300 °C for 2 h or solution heat treated at 530 °C for 6 h in air, followed by water-quench to room temperature. Note that thermodynamic calculations [25,29] have been accessed that the different temperatures of 300 °C and 530 °C correspond to a four-phase region of α-Al + Si (diamond) + β-AlFeSi (τ_6_-Al_9_Fe_2_Si_2_ [30,31]) + Mg_2_Si and a three-phase region of α-Al + Si (diamond) + β-AlFeSi, respectively.

The cross sections (parallel to the Z-direction) of specimens for optical microscopic observation were mechanically polished and subsequently finished by electropolishing. Perchloric acid-ethyl alcohol mixture with a volume ratio of 9:1 was used for the electropolishing at room temperature. The cross sections of specimens for observation by field-emission type scanning electron microscope (FE-SEM, JEOL JSM-7401, Tokyo, Japan) were ion-milled by using a JEOL cross section polisher. The orientation distribution of the α-Al (fcc) matrix was conducted by electron backscatter diffraction (EBSD) analyses operated at an acceleration voltage of 20 kV and a step size of 2 µm. The specimens for transmission electron microscopic (TEM, JEOL JEM-2100Plus, Tokyo, Japan) observations were cut into thin foils and then prepared by using an ion slicer at 6 V. The TEM observations were performed by using JEOL JEM-2100 plus equipped with energy-dispersive X-ray spectroscopy (EDS) operated at 200 kV. The thermodynamic equilibrium calculation for the Al–Si–Mg–Fe quaternary system was performed for the studied alloy composition of Al–10.23Si–0.37Mg–0.12Fe (wt %) [22], using a thermodynamic database for the Al-based multicomponent system (PanAl) [32]. The calculation was utilized to quantify volume fractions of constituent phases in the studied alloy composition in equilibrium according to the previous reports [25,29]. The area fraction and size of Si particles were quantified by more than five SEM images using an image analysis software (Image J, National Institutes of Health, Madison, WI, USA). Vickers hardness tests were carried out for the mechanically polished specimens at room temperature at a load of 0.98 N and for a load holding time of 15 s. More than five indentation tests were performed on each location of the specimens.

## 3. Results

### 3.1. Node Portions in Lattice Structure

**Figure 2:**
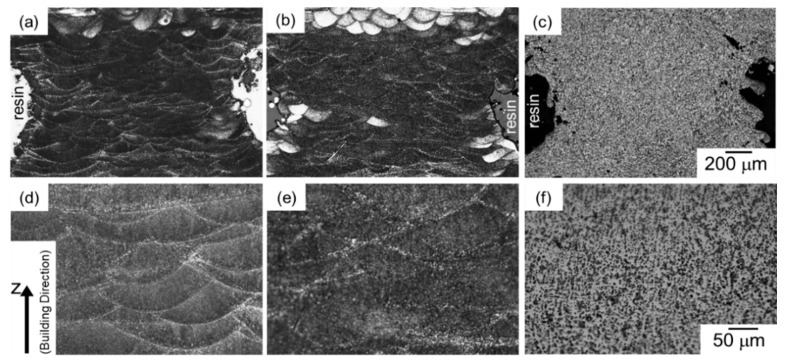
Optical micrographs of microstructures taken from the node portion of the lattice specimen at (**a**–**c**) low magnification and (**d**–**f**) high magnification: (**a**,**d**) as-manufactured, (**b**,**e**) annealed at 300 °C for 2 h, and (**c**,**f**) solution heat treated at 530 °C for 6 h.

Figure 2 presents optical micrographs of the microstructures taken from the node portions of AlSi10Mg lattice specimens: (a,d) as-manufactured specimen, (b,e) 300 °C annealed specimen, and (c,f) 530 °C solution-treated specimen. In the as-manufactured specimen (Figure 2a,d), microstructure appears to be composed of a number of melt pools. This microstructural characterization corresponds with the local melting and rapidly solidifying regions by the scanning laser irradiation in the LPBF process. The melt pools were observed in the specimen annealed at 300 °C as well (Figure 2b,e), whereas their morphologies were not observed in optical micrographs of 530 °C solution treated specimen (Figure 2c,f). The change in microstructure observed by optical microscope with the annealing at elevated temperatures corresponds well to that of the bulk specimens manufactured via LPBF process [25].

**Figure 3:**
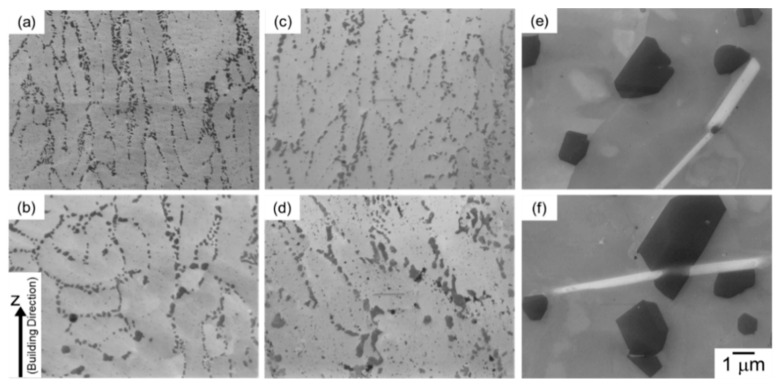
SEM micrographs of the microstructures taken from the node portion of the lattice specimen with different vertical distances from the geometric center: (**a**,**c**,**e**) *d_N_* = 0.8 mm and (**b**,**d**,**f**) *d_N_* = −0.8 mm; (**a**,**b**) as-manufactured, (**c**,**d**) annealed at 300 °C for 2 h, and (**e**,**f**) solution heat treated at 530 °C for 6 h.

Figure 3 displays SEM micrographs of microstructures taken from different locations in the node portions of as-manufactured and heat treated specimens. The as-manufactured specimen exhibits a cellular structure composing of primary α-Al phases bounded by a network of fine α-Al/Si eutectic microstructure. A number of elongated α-Al phases with a mean width (*w*_α_) of approximately 0.8 µm were observed at the upper location of the node portion (*d_N_* = 0.8 mm, Figure 3a), whereas relatively equiaxed and coarser α-Al phases (above 2 µm) were observed at the lower location (*d_N_* = −0.8 mm, (Figure 3b)). These results indicate the gradient microstructure formed in the node portions of the LPBF-manufactured AlSi10Mg lattice specimen, as described in detail elsewhere [22]. After the annealing at 300 °C for 2 h (Figure 3c,d), the observed cellular structure became coarsened and fine Si particles precipitated within the primary α-Al phases. The difference in microstructure at between upper and lower portions (corresponding to the continuous microstructural changes) was found in the lattice specimen annealed at 300 °C as well. After the solution heat treatment at 530 °C for 6 h (Figure 3e,f), the observed microstructure changed significantly. Significantly coarsened Si particles and Fe-rich intermetallic phases with a rod-shape morphology were observed in the α-Al matrix. The coarsened microstructure was observed in both of the upper (*d_N_* = 0.8 mm, Figure 3e) and lower (*d_N_* = −0.8 mm, Figure 3f) locations, indicating a homogenous microstructure developed in the lattice specimen solution treated at an elevated temperature of 530 °C.

**Figure 4:**
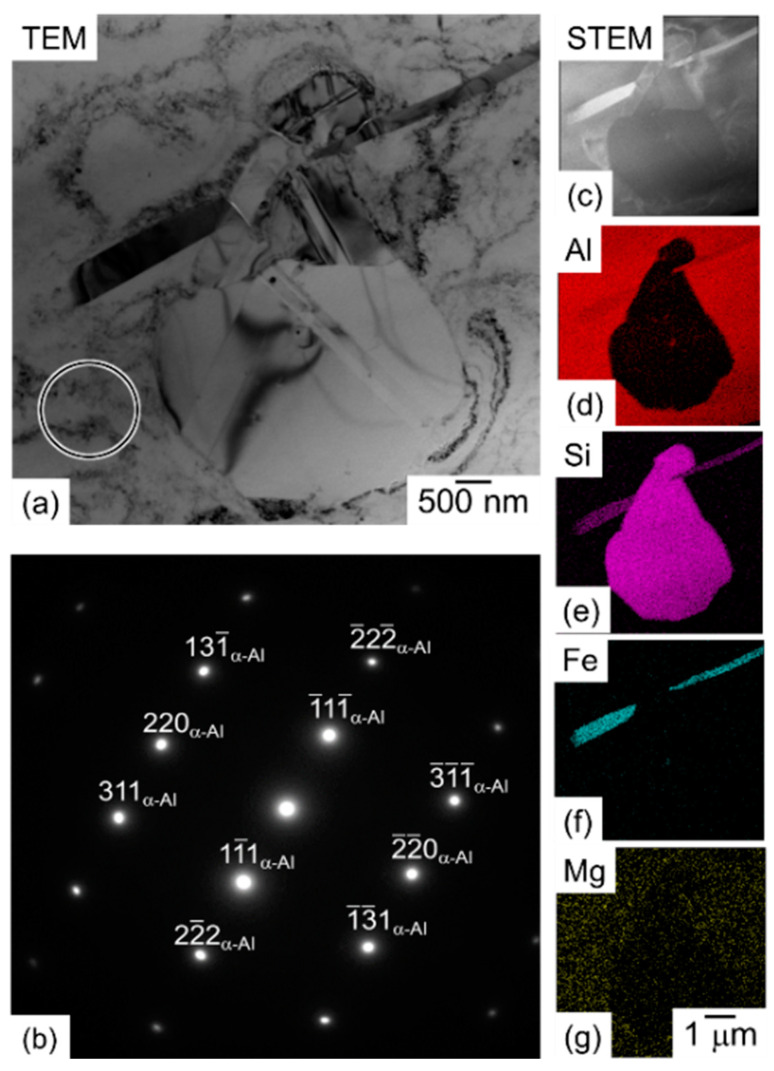
(**a**) TEM bright-field image of the microstructure taken from the node portion of the lattice specimen solution treated at 530 °C for 6 h with the vertical distance from the geometric center: *d_N_* = 0.8 mm. (**b**) Selected area electron diffraction (SAED) patterns from the region marked with a circle in (**a**). (**c**) STEM-HAADF (scanning transmission electron microscopic high-angle annular dark-field) image and (d–g) the corresponding EDS elemental mapping images of (**d**) Al, (**e**) Si, (**f**) Fe, and (**g**) Mg.

Figure 4 displays the result of TEM characterizations for the node portion in the lattice specimen solution treated at 530 °C for 6 h. A relatively coarsened Si particle with a size of approximately 4 µm and an intermetallic phase with a rod-shaped morphology (dark contrast) were observed in a TEM bright-field image (Figure 4a). The selected area electron diffraction (SAED) patterns (Figure 4b) show the α-Al matrix with fcc structure, indicating that any fine Si particles were not precipitated in the α-Al matrix. The EDS chemical analysis revealed the observed intermetallic phases contain Al, Si, and Fe elements (Figure 4c–g), which is indicative of β-AlFeSi (τ_6_-Al_9_Fe_2_Si_2_ [30,31]) intermetallic phase formed [25]. Note that Mg element was detected inside the α-Al matrix in the 530 °C solution treated specimen (Figure 4g).

**Figure 5:**
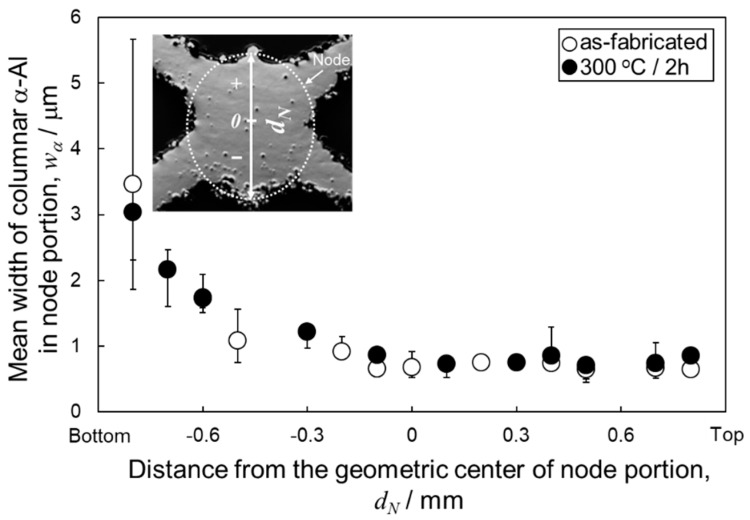
Changes in the mean width of columnar α-Al phases as a function of the vertical distance from the geometric center (*d_N_*) in the node portion of as-manufactured and 300 °C annealed specimens.

**Figure 6:**
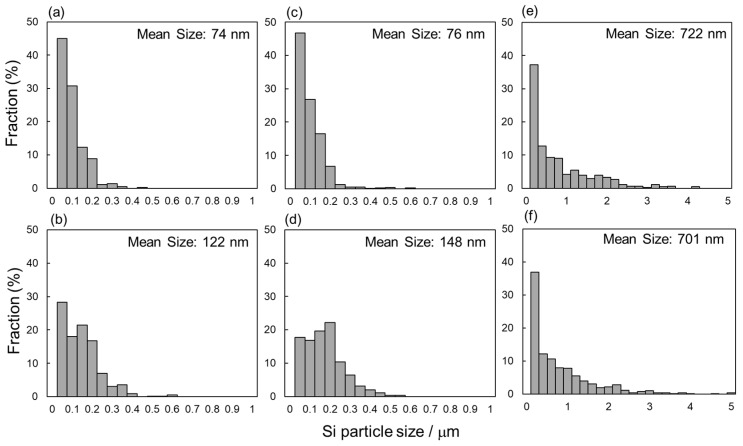
Si particle size distribution measured from the node portion of the lattice specimen with different vertical distances from the geometric center: (**a**,**c**,**e**) *d_N_* = 0.8 mm, (**b**,**d**,**f**) *d_N_* = −0.8 mm; (**a**,**b**) as-manufactured, (**c**,**d**) annealed at 300 °C for 2 h, and (**e**,**f**) solution heat treated at 530 °C for 6 h.

Figure 5 shows the changes in the mean width of columnar α-Al phases as a function of the vertical distance from the geometric centers of the node portions. The measured values of as-manufactured and 300 °C annealed specimens were plotted in this figure. The widths of α-Al phases were almost constant at approximately 0.8 µm at the upper and center locations (*d_N_* = 0.8~−0.1 µm), whereas it increased with decreasing *d_N_* at the lower location (*d_N_* < −0.1 mm). These results indicate the gradient microstructures in both as-manufactured and 300 °C annealed specimens. Figure 6 presents the Si particle size distributions measured from the node portion. In the as-manufactured specimen, the average sizes of Si particles at the upper (*d_N_* = 0.8 mm, Figure 6a) and lower (*d_N_* = −0.8 mm, Figure 6b) locations are 74 nm and 122 nm, respectively. The Si particles with a larger average size observed at the lower location correspond well to the relatively coarsened microstructure observed (Figure 3b). After the annealing at 300 °C for 2 h, the average sizes of Si particles slightly increased to 76 nm and 148 nm at the upper (*d_N_* = 0.8 mm, Figure 6c) and lower (*d_N_* = −0.8 mm, Figure 6d) locations, respectively. The Si particle size significantly increased to approximately 700 nm at both upper and lower locations after the solution heat treatment at 530 °C (Figure 6e,f), corresponding to the homogenous microstructure observed by SEM (Figure 3e,f).

**Figure 7:**
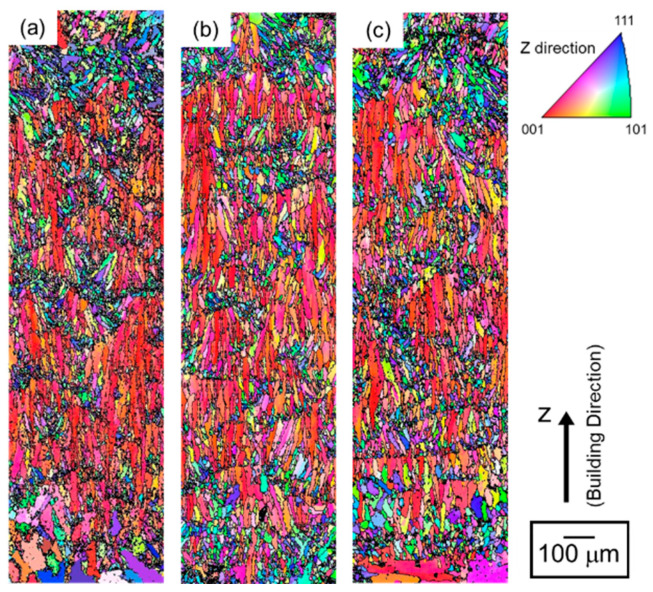
Orientation distribution maps of the node portion of the lattice specimen: (**a**) as-manufactured, (**b**) annealed at 300 °C for 2 h, and (**c**) solution heat treated at 530 °C for 6 h.

**Figure 8:**
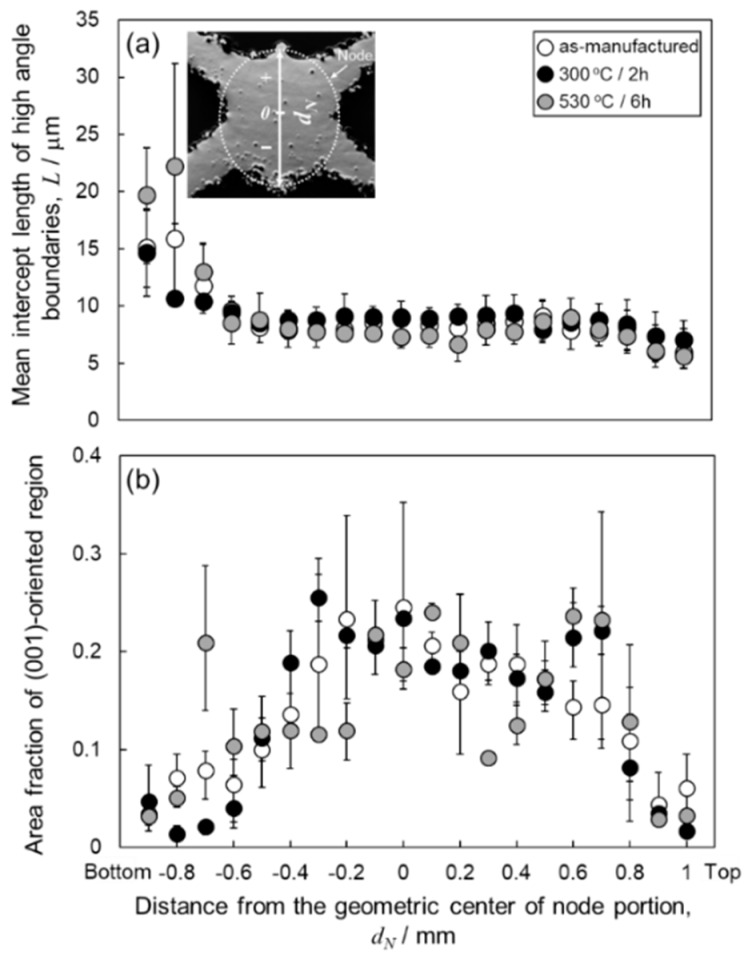
Change in (**a**) mean intercept length of high-angle boundaries and (**b**) area fraction of the (001)-oriented region plotted with vertical distance from the geometric center (*d_N_*) of the node portion of the lattice specimen with heat treatment.

Figure 7 depicts the orientation distribution maps of the α-Al (fcc) phase in the node portions. The colors represent the orientation along the building direction (Z-direction) according to the orientation color key in the unit triangle. All the specimens present a similar microstructure of the α-Al matrix mostly composed of elongated grains along the Z-direction. Equiaxed fine grains were often observed near melt pool boundaries. Most of the elongated grains were shown the <001> orientation along the Z-direction (red areas). Coarser α-Al grains were localized close to the bottom surface (at the lower location) corresponding to *d_N_* < −0.5 mm. To quantify the change in the microstructure of α-Al phase with annealing, the mean intercept length of high-angle boundaries (*L*) was evaluated using the EBSD analyzed data. According to quantitative microscopy [33], the mean intercept length of high angle boundaries (*L*) can be used to evaluate the mean grain size. The *L* values plotted with *d_N_* are presented in Figure 8a. In all the specimens, the *L* values slightly increase with decreasing *d_N_* until *d_N_* = 0.8 mm. The *L* values become almost constant of approximately 8 µm from *d_N_* = 0.8 mm to *d_N_* = −0.5mm. It is noteworthy that the *L* values increase to approximately 15–20 µm at the locations from *d_N_* = −0.6 mm to *d_N_* = −0.9 mm (the lower location), which corresponds well to the coarsened microstructure observed around the bottom surface (Figure 7).

To identify the evolution of the crystallographic texture with heat treatment, the area fraction of the <001>-oriented grains along the Z-direction was evaluated by using EBSD analyses. In the present analyses, the tolerance misorientation angle of 15° from the <001> direction has been set. The results are shown in Figure 8b. In all specimens, low area fractions of the <001>-oriented grains are approximately 5% at the upper locations (*d_N_* > 0.8 mm) and increase with decreasing *d_N_*. At the locations close to the geometric center of the node portions (−0.3 mm < *d_N_* < 0.8 mm), the area fractions increased to approximately 20%. The area fraction decreases at the lower locations (*d_N_* < −0.3 mm), corresponding to the coarsened grains (Figure 7) with different orientations. These results indicate that the crystallographic textures remain unchanged after the heat treatment at elevated temperatures of 300 °C and 530 °C.

### 3.2. Strut Portions in Lattice Structure

**Figure 9:**
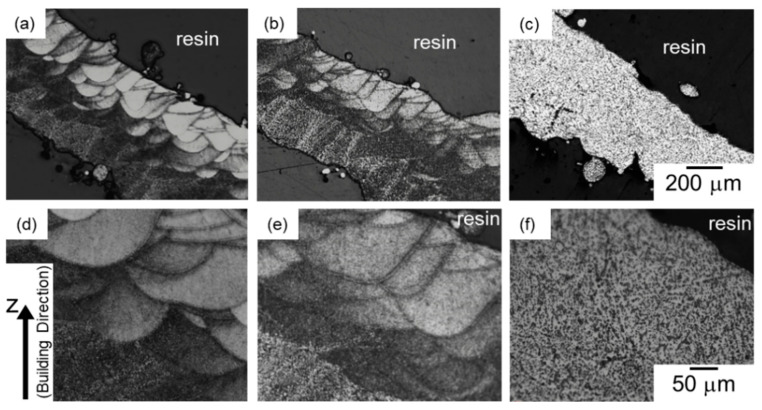
Optical micrographs of microstructures taken from the strut portion of the lattice specimen at (**a**–**c**) low magnification and (**d**–**f**) high magnification: (**a**,**d**) as-manufactured, (**b**,**e**) annealed at 300 °C for 2 h, and (**c**,**f**) solution heat treated at 530 °C for 6 h.

Figure 9 presents optical micrographs of the microstructures taken from the strut portions of the electropolished specimens. A similar trend of a change in microstructure by heat treatment was found in the strut portions as well as the node portions. Microstructure composed of several melt pools was observed in the strut portions of the as-manufactured specimen (Figure 9a,d). The microstructural morphology was observed in the 300 °C annealed specimen (Figure 9b,e), whereas it was not recognized in the 530 °C solution treated specimen (Figure 9c,f).

**Figure 10:**
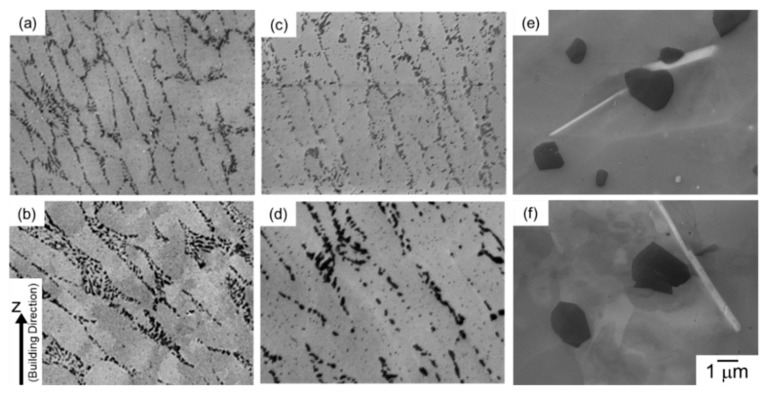
SEM micrographs of the microstructures taken from the strut portion of the lattice specimen with different vertical distances from the geometric center: (**a**,**c**,**e**) *d_S_* = 0.2 mm and (**b**,**d**,**f**) *d_S_* = −0.3 mm; (**a**,**b**) as-manufactured, (**c**,**d**) annealed at 300 °C for 2 h, and (**e**,**f**) solution heat treated at 530 °C for 6 h.

Figure 10 presents SEM micrographs of microstructures taken from different locations in the strut portions of the as-manufactured specimen and annealed specimens. The cellular structure composing of elongated α-Al phases bounded by α-Al/Si eutectic microstructure was observed in the strut portion of the as-manufactured specimen as well (Figure 10a,b). The α-Al phases with a mean width (*w*_α_) of approximately 0.8 µm are elongated along the building direction (Z-direction) at the upper location (Figure 10a), whereas the elongated direction of α-Al phases appears to be along the inclined direction of the strut portion at the lower location, as shown in Figure 10b. Relatively coarser α-Al phases (approximately 1.5 µm) were observed at the lower location of strut portions (Figure 10b). After the annealing at 300 °C for 2 h (Figure 10c,d), these microstructural features changed slightly, whereas fine Si particles were precipitated within the columnar α-Al phases. After the solution heat treatment at 530 °C (Figure 10e,f), the observed cellular structure changed to coarsened Si particles and intermetallic phases (®-AlFeSi) formed in the α-Al matrix. The coarsened microstructure was found at both of upper (*d_S_* = 0.2 mm, Figure 10e) and lower (*d_S_* = −0.3 mm, Figure 10f) locations, which is indicative of the formation of homogenous microstructure in the strut portions at an elevated temperature of 530 °C.

**Figure 11:**
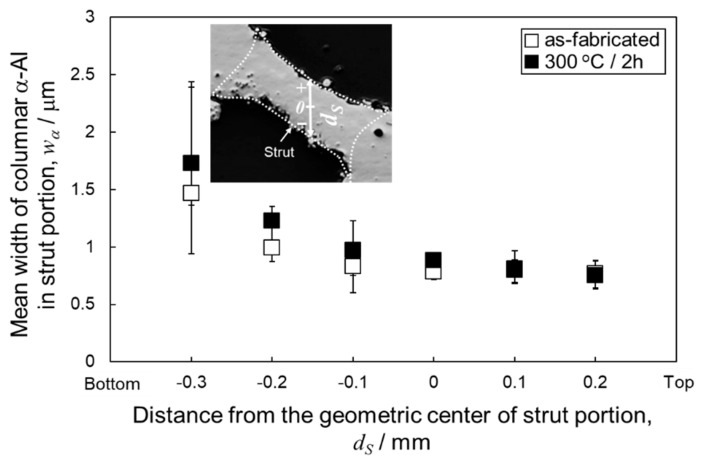
Changes in the mean width of columnar α-Al phases as a function of the vertical distance from the geometric center (*ds*) in the strut portion of as-manufactured and 300 °C annealed specimens.

**Figure 12:**
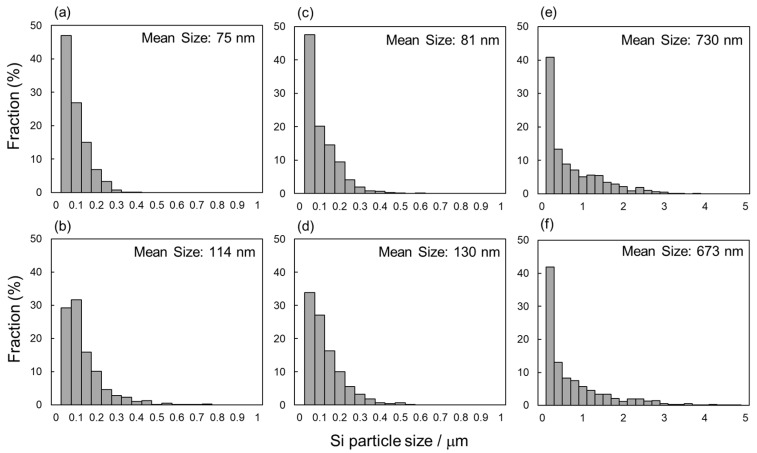
Si particle size distribution measured from the strut portion of the lattice specimen with different vertical distances from the geometric center: (**a**,**c**,**e**) *d_S_* = 0.2 mm, (**b**,**d**,**f**) *d_S_* = −0.3 mm; (**a**,**b**) as-manufactured, (**c**,**d**) annealed at 300 °C for 2 h, and (**e**,**f**) solution heat treated at 530 °C for 6 h.

Figure 11 presents the changes in the mean width of columnar α-Al phases as a function of the vertical distance from the geometric centers of strut portions in as-manufactured and 300 °C annealed specimens. The *w*_α_ value was almost constant at approximately 0.8 μm from *d_S_* = 0.2 μm to *d_S_* = −0.1 μm, whereas it increased with decreasing *d_S_* at the lower location (*d_S_* < −0.1 mm), indicating a continuous change in microstructure inside the strut portions of both as-manufactured and 300 °C annealed specimens. Notably, the coarsened α-Al phase observed at the locations near the bottom surface of the node portion (Figure 5, *d_N_* <−0.6 mm) appears pronounced in comparison with the strut portion. Figure 12 exhibits the Si particle size distribution measured from the strut portion. In the as-manufactured specimen, the average Si particle size at the upper (*d_S_* = 0.2 mm, Figure 12a) and lower (*d_S_* = −0.3 mm, Figure 12b) locations were 75 nm and 114 nm, respectively. After the annealing at 300 °C for 2 h, the average Si particle size slightly increased to 81 nm and 130 nm at the upper (*d_S_* = 0.2 mm, Figure 12c) and lower (*d_S_* = −0.3 mm, Figure 12d) locations, respectively. After the solution heat treatment at a higher temperature of 530 °C for 6 h, the average Si particle sizes significantly increased to 730 nm and 673 nm at the upper (*d_S_* = 0.2 mm, Figure 12e) and lower (*d_S_* = −0.3 mm, Figure 12f) locations, respectively. These changes in Si particle size observed in the strut portions by heat treatment are similar to those in the node portions (Figure 6).

**Figure 13:**
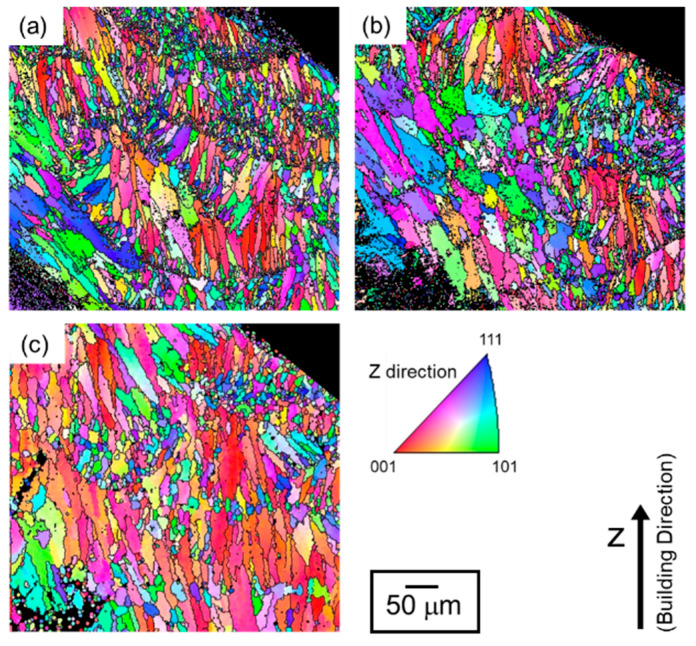
Orientation distribution maps of strut portion of the lattice specimen: (**a**) as-manufactured, (**b**) annealed at 300 °C for 2 h, and (**c**) solution heat treated at 530 °C for 6 h.

**Figure 14:**
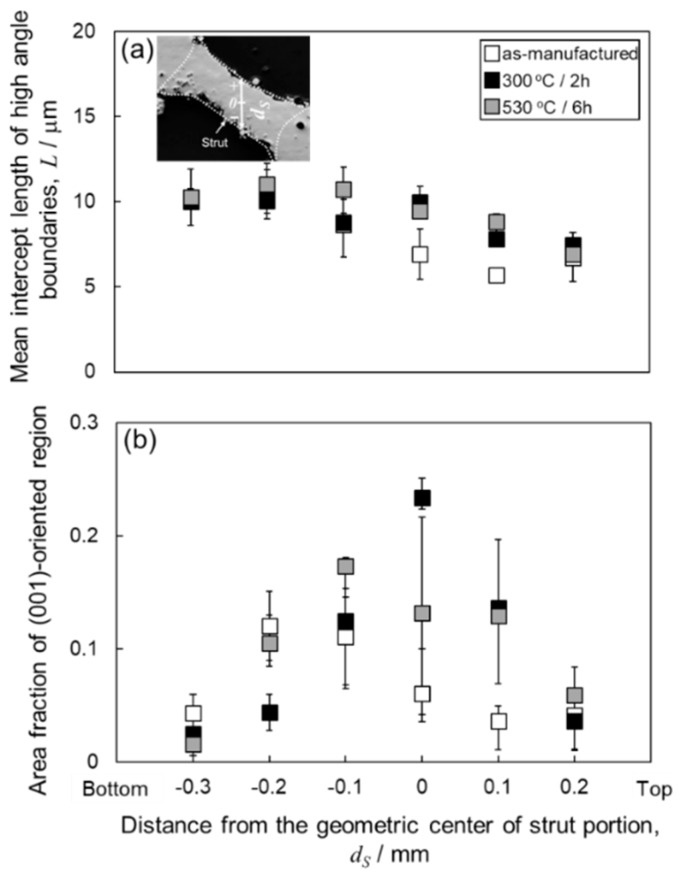
Changes in (**a**) mean intercept length of high-angle boundaries and (**b**) area fraction of the (001)-oriented grains plotted with vertical distance from the geometric center (*d_S_*) of the strut portion of the lattice specimen with heat treatment.

Figure 13 present the orientation distribution maps of the α-Al (fcc) phase in the strut portions. The colors in the orientation distribution maps represent the orientation along the building direction (Z-direction) according to the orientation color key in the unit triangle. The observed microstructures of α-Al matrix consist of a number of elongated grains in the strut portion. At the locations close to bottom surfaces (*d_S_* < 0 mm), the elongated direction of the grains is found to parallel to the direction of the strut portion. The <001>-oriented grains (red areas) were scarcely observed close to the bottom surface of the strut portion (*d_S_* < 0 mm). These crystallographic features were found in all specimens, whereas the equiaxed grains often coarsened localized at melt pool boundaries after the solution heat treatment at 530 °C (Figure 13c). The results of mean intercept length of high-angle boundaries (*L*) measured in the strut portions are exhibited in Figure 14a. A trend can be found that, in all specimens, the *L* value increases to approximately 10 μm with decreasing *d_S_*. Figure 14b shows the variations of area fractions of the <001>-oriented grains measured in the strut portions. The area fractions of <001>-oriented grains at locations close to the top (*d_S_* > 0.1 mm) and bottom (*d_S_* < −0.2 mm) surfaces were significantly lower than that at locations close to the geometric center (−0.2 mm < *d_S_* < 0.1 mm) of the strut portion. The variation of <001>-oriented grains depending on the locations slightly changes by annealing at elevated temperatures. The similar trends were found in the node portion as well (Figure 8b).

### 3.3. Microhardness

**Figure 15:**
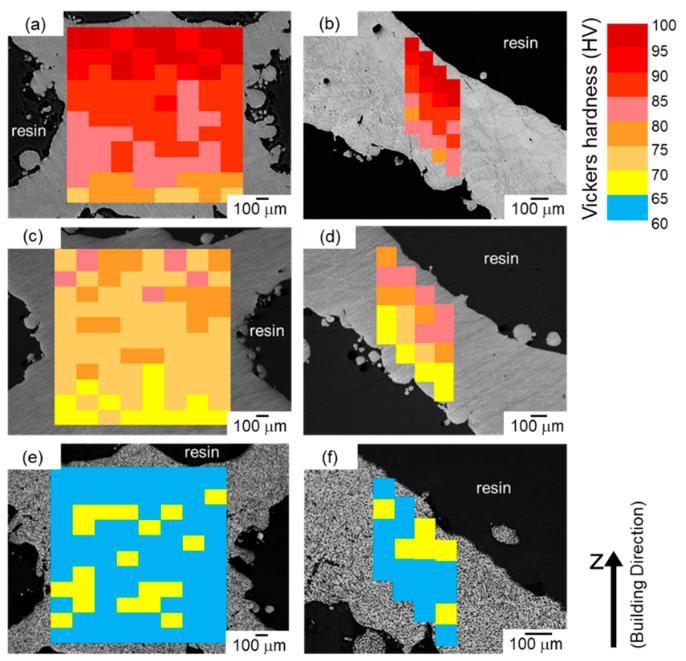
Microhardness maps indicating local hardness of various areas within (**a**,**c**,**e**) node portion and (**b**,**d**,**f**) strut portion of the lattice specimen; (**a**,**b**) as-manufactured, (**c**,**d**) annealed at 300 °C for 2 h, and (**e**,**f**) solution heat treated at 530 °C for 6 h.

Figure 15 presents the microhardness map within the node and strut portions of studied specimens. In the node and strut portions, the microhardness was reduced by the annealing and its reduction was more pronounced at the higher temperature. In the node potion (Figure 15a,b) of the as-manufactured specimen, the microhardness was measured to approximately 100 HV at the upper location (corresponding to *d_N_* = 1 mm), whereas the microhardness gradually decreased with approaching the bottom surface (corresponding to decreasing *d_N_* value) and became below 80 HV around the bottom surface (corresponding to *d_N_* = −0.8 mm). After annealing at 300 °C (Figure 15c,d), the microhardness was reduced at all locations, whereas a gradient change in the measured microhardness depending on the location can be found as well. The varied microhardness depending on the location corresponds well to the change in microstructural parameters such as the mean width of elongated α-Al phase, mean width of columnar α-Al phases, *w*_α_ (Figure 5) and the grain size, *L* (Figure 7a). In the specimen solution treated at 530 °C, the measured microhardness was almost constant of approximately 62 HV independent of the location (*d_N_* value), indicating the formation of homogenous microstructure inside the node portions. The similar trend of the varied microhardness depending on the location was observed in the strut portion (Figure 15b,d,f)). In the as-manufactured specimen, the microhardness gradually decreased with approaching the bottom surface (corresponding to reducing *d_N_* value) of the strut portion, whereas the microhardness appeared constant of approximately 60 HV in the 530 °C solution treated specimen. The abovementioned results of the measured microhardness were consistent with the changes in microstructural parameters of *w*_α_ and *L* depending on the location of both node (Figure 5 and Figure 7a) and strut portions (Figure 9 and Figure 12a).

## 4. Discussion

**Figure 16:**
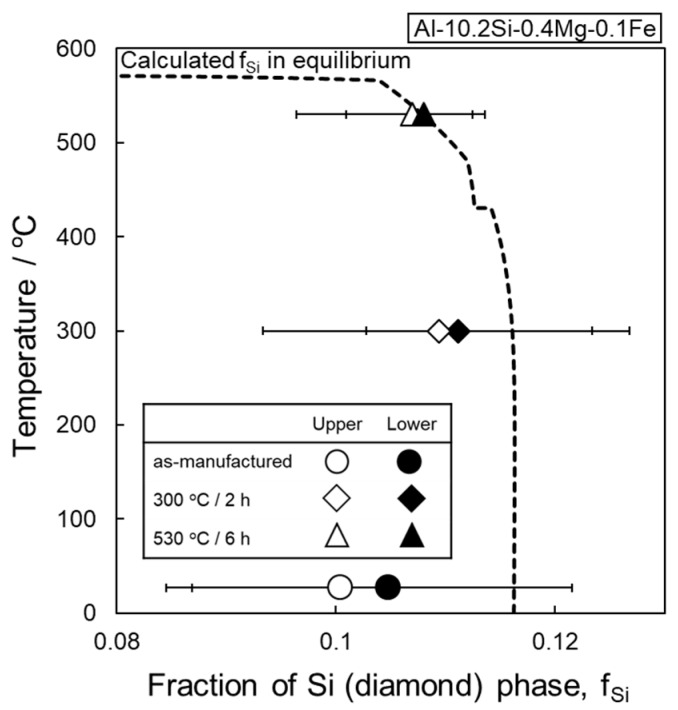
Experimentally measured area fraction of Si particles in node portion of the lattice specimen plotted with annealing temperature, comparing with the calculated volume fraction of Si particles in the studied alloy composition in equilibrium.

This study proved the coarsening of microstructure and its associated low hardness of the AlSi10Mg lattice specimen heat treated at elevated temperatures. The present microstructural characterizations confirmed the gradient microstructure (continuous microstructural changes) developed in the node and strut portions of as-manufactured AlSi10Mg lattice specimen. The inhomogeneous microstructure was observed in lattice structures of other alloys (Cu, Co, stainless steel) manufactured via LPBF as well [34,35,36]. The heat treatment process enhances the microstructural coarsening resulting in reduced hardness. The continuous microstructural change and its related variation of measured hardness still remain after the annealing at 300 °C, whereas the coarsening of Si particles is much pronounced at a higher temperature of 530 °C, leading to a homogenous coarsened microstructure in all portions of the AlSi10Mg lattice specimen. One of the important findings to understand the microstructural coarsening is that fine Si particles precipitate within the α-Al phase at an elevated temperature of 300 °C. The observation indicates a supersaturated solid solution of α-Al phase containing Si in solution formed in the as-manufactured AlSi10Mg lattice specimen. Liu et al. proposed the formation mechanism of the gradient microstructure [22]. It could form at varied cooling rates depending on the location associated with the built surfaces due to the interface between solidified and alloy powder (unmelted) regions with low thermal conductivity. The proposed mechanism is indicative of different contents of solute Si depending on the location inside the as-manufactured AlSi10Mg lattice specimen. In order to quantify the solute Si in the α-Al matrix and its change with the heat treatment process, the area fractions of the Si particles were measured by analyzing a couple of SEM images observed in studied specimens and compared with the calculated volume fraction of Si particles in equilibrium. The results are presented in Figure 16. The measured area fractions of the as-manufactured specimens were plotted at ambient temperature (27 °C). The average measured area fractions at the upper and lower locations in the node portion of the as-manufactured specimen were approximately 10.0% and 10.5%, respectively. Both values were lower than the calculated one in equilibrium (11.6%) at ambient temperature, indicating the α-Al matrix containing solute Si in supersaturation. The lower area fraction of Si particles at the upper location showed reasonably good agreement with the observed fine microstructures comparing with those observed at the lower location (Figure 3). The average measured fractions became higher to approximately 11% after the annealing at 300 °C, corresponding to the Si particles finely precipitated within the columnar α-Al phase (Figure 3c,d). After the solution heat treatment at 530 °C, the area fractions measured at both locations became 10.7%, which corresponded to the calculated one in equilibrium at 530 °C. This result obviously indicated that a microstructural equilibrium was almost reached after the solution heat treatment at 530 °C for 6 h, which was consistent with the formation of Fe-rich intermetallic phase (Figure 4) assessed in the calculated phase diagram [28]. The aforementioned change in solute Si in the α-Al matrix is in good agreement with the results of XRD analysis in previous studies [24,26]. These results represent the heat treatment process could facilitate microstructural coarsening reaching an equilibrium state, resulting in the formation of homogenous microstructure in the annealed AlSi10Mg lattice specimen. Note that the effect of residual stress on the microstructural change by the heat treatments would be negligible considering the low stress level (below 25 MPa) measured in the AlSi10Mg specimens manufactured via LPBF using the pre-heating base plate [37,38,39].

Recently, Suzuki et al. demonstrated that the compressive deformation behavior of the AlSi10Mg lattice structure was improved to achieve a stable compressive deformation by subsequent heat treatments [23]. One of the possible reasons for the unstable compressive behavior of the as-manufactured AlSi10Mg lattice structure is a localized deformation enhanced by gradient microstructure inside various structural portions. It can be supposed that the localized strain would lead to the occurrence of macroscopic shear in the lattice structure under compression. The present study demonstrated a homogenous microstructure in the 530 °C solution treated AlSi10Mg lattice specimen, whereas it exhibits the unstable compressive stress as well. These results confirmed that the microstructural inhomogeneity could have a slight influence on achieving the stable compressive stress of the lattice structure. Therefore, a dominant contributor to control the compressive properties would be microstructure itself and its associated ductility of all portions composed of the AlSi10Mg lattice structure. It has been found that the 300 °C annealed lattice specimen exhibits relatively stable plateau stress. The 300 °C annealed specimen exhibits an adequate tensile ductility attributed to a large post-uniform elongation [25]. The unique ductility may suppress the localized deformation inside the strut portions contributing to the macroscopic shear occurring in the lattice structure, whereas the detailed mechanisms of the tensile ductility sill remain unclear. In order to control the stable compressive stress of the AlSi10Mg lattice structure by heat treatment processes, it is required to further understand the relation between microstructural parameters and ductility of the LPBF-manufactured AlSi10Mg specimens and its change by heat treatments.

## 5. Conclusions

The present study systematically demonstrated the effect of heat treatment process on microstructure of the AlSi10Mg lattice structure manufactured via LPBF. The main results and findings are summarized as follows.

(1) The as-manufactured lattice specimen exhibited a characteristic cellular structure composing of primary α-Al phase bounded by α-Al/Si eutectic microstructure. A gradient microstructure was found at the node and strut portions of the lattice specimen. The microstructure appears more equiaxed and coarser with approaching the bottom surface of both portions.

(2) At an elevated temperature of 300 °C, the fine Si particles precipitate within the primary α-Al phase and eutectic Si particle coarsening occur. The microstructural coarsening is more pronounced at a higher temperature. At an elevated temperature of 530 °C, the gradient microstructure was not observed. The homogenous microstructure consisting of significantly coarsened Si particles and a stable Fe-containing intermetallic phase (β-AlFeSi) were observed at all locations.

(3) The gradient microstructure found in as-manufactured and 300 °C annealed specimens contributed to a variation in hardness measured at different locations in the lattice specimen. At an elevated temperature of 530 °C, the homogeneous microstructure formed, leading to homogeneity in hardness at all locations. The homogenization of mechanical properties in the lattice specimens has been approached at this temperature.

## Figures and Tables

**Table 1:** The process parameters used for manufacturing AlSi10Mg alloy specimens.

Process Parameters	Values
Laser power (W)	370
Scanning speed (mm/s)	1300
Spot size (µm)	100
Hatch distance (µm)	70
Layer thickness (µm)	30
Volumetric energy density (J∙mm^−3^)	136
